# Effect of traditional Chinese medicine on sleep and blood pressure in patients with comorbid hypertension-insomnia: a systematic review and meta-analysis

**DOI:** 10.3389/fpsyt.2026.1882984

**Published:** 2026-09-03

**Authors:** Yuchen Fan, Haijun Li, Yuezhen Ma, Shuangshuang Yin, Cuiping Xu

**Affiliations:** 1 School of Traditional Chinese Medicine, Shandong University of Traditional Chinese Medicine, Jinan, Shandong, China; 2 Department of Nephrology and Rheumatology, Shandong Provincial Third Hospital, Shandong University, Jinan, China; 3 Department of Nursing, Shandong Provincial Third Hospital, Shandong University, Jinan, China; 4 Department of Nursing, The First Affiliated Hospital of Shandong First Medical University & Shandong Provincial Qianfoshan Hospital, Jinan, China

**Keywords:** cardiovascular diseases, circadian rhythm, hypertension, sleep disorders, sleep quality, traditional Chinese medicine

## Abstract

**Introduction:**

Hypertension and insomnia frequently occur together and share neurophysiological and vascular regulatory disturbances, which increase the risk of cardiovascular disease. Traditional Chinese Medicine (TCM) uses syndrome differentiation to guide the therapeutic selection for sleep disturbance and blood pressure regulation. This study evaluated whether syndrome-informed TCM improves the sleep quality and blood pressure in adults with comorbid hypertension and insomnia.

**Methods:**

We systematically searched databases including PubMed/MEDLINE, EMBASE, and Scopus from 2013 to 2025. Randomized controlled trials (RCTs) with syndrome-classified TCM interventions that reported sleep or blood pressure outcomes were eligible for the review. The sleep outcomes measured with the Pittsburgh Sleep Quality Index or Insomnia Severity Index were pooled as standardized mean differences, while systolic and diastolic blood pressure measures were synthesized separately.

**Results:**

From 940 screened records, 16 studies met the inclusion criteria, and 12 trials provided data suitable for the quantitative synthesis. Quantitative synthesis showed a significant improvement in sleep quality with syndrome-informed TCM (SMD −2.21, 95% CI −3.26 to −1.16). Blood pressure reduction was smaller but statistically significant (SMD −0.43, 95% CI −0.64 to −0.21), with systolic reductions ranging from 2.5 to 6.2 mmHg in hypertensive cohorts.

**Conclusion:**

The RCT analysis showed greater sleep improvement with TCM in patients with defined syndrome eligibility, with mild adverse events and outcome variation related to syndrome criteria, intervention modality, and treatment duration. Hence, the findings indicate a marked improvement in sleep and a reduction in blood pressure with syndrome-informed TCM.

**Systematic Review Registration:**

https://www.crd.york.ac.uk/PROSPERO/, identifier CRD420261468564.

## Introduction

Hypertension and insomnia are frequently observed within the same adult population and are associated with increased cardiovascular risk and reduced quality of life. Population-based data have shown that 19% to 47.9% of individuals diagnosed with hypertension experience clinically significant insomnia, and adults with chronic insomnia show a 30% to 50% higher probability of developing hypertension compared with individuals without sleep disturbance (1–3). A recent cross-sectional study from rural eastern India reported poor sleep quality (PSQI >7) in 48.2% of adults with hypertension. Mean PSQI scores were significantly higher in hypertensive individuals than in normotensive individuals (11.56 ± 3.4 vs. 4.78 ± 2.6; P = 0.001). The highest odds of poor sleep quality were observed in physically inactive adults and those who were overweight or underweight (4). The biological basis involves shared pathophysiological mechanisms. The dysregulation of the sympathetic nervous system, hypothalamic-pituitary-adrenal axis, and endothelial function is observed in both hypertension and insomnia, with impaired sleep quality and elevated blood pressure aggravating these abnormalities. Antihypertensive and sedative-hypnotic medications may cause dependence, cognitive adverse effects, and short-term benefit, thereby increasing the interest in other non-pharmacological methods (1, 3).

Clinical studies have demonstrated associations between TCM constitutional classification, sleep regulation, and cardiovascular control in patients with hypertension and insomnia (5–9). These findings validate the use of acupuncture and related traditional therapies for management of both conditions, with several differences in outcomes according to syndrome type (10–16). The diagnosis of TCM depends on syndrome differentiation, which identifies clinical entities like liver yang hyperactivity, heart-kidney disharmony, qi stagnation with phlegm heat, and blood deficiency with spirit disturbance (17–19). Treatment is selected according to the diagnosed syndrome and includes herbal medicines and acupuncture. Jiaotaiwan is prescribed for heart-kidney disharmony (20), Xiao Yao San for liver qi stagnation associated with insomnia and emotional symptoms (21), and Suanzaoren Tang for heart blood deficiency (15). Acupuncture prescriptions are also syndrome-directed. Frequently used acupoints include HT7 (Shenmen), KI3 (Taixi), LR3 (Taichong), and DU20 (Baihui), which help to improve sleep disturbance and related clinical manifestations (1, 10, 22).

The 2024 meta-analysis showed that acupuncture reduced Pittsburgh Sleep Quality Index (PSQI) scores (MD −5.71, 95% CI −8.19 to −3.23) and lowered systolic blood pressure in adults with hypertension and insomnia (1). Herbal formulas Yangxue Qingnao Pills, indicated for blood deficiency with gan yang hyperactivity, and Huoxue Qianyang Qutan Recipe showed blood pressure control comparable to losartan and also improved sleep related and emotional symptoms (23, 24). The safety analyses across trials showed low rates of adverse events and improvements in health-related quality of life, as measured using the SF-36 (25, 26). The experimental literature describes effects on oxidative stress regulation, autonomic nervous system activity, and gut microbiota-brain pathways that are relevant to both hypertension and insomnia (9, 27).

The existing systematic reviews commonly combine diverse TCM interventions without stratification by syndrome classification, which limits evaluation of pattern-specific effects (7, 28, 29). The recent randomized trial by Xiong et al. demonstrated greater improvement with pattern-based herbal prescriptions compared with fixed formulas in primary insomnia (17). The population studies also show associations between specific TCM constitutions, including qi deficiency and qi stagnation, and insomnia severity (6, 19, 30).

The clinical interaction between hypertension and insomnia and the use of pattern-based treatment in TCM indicate a need for structured evidence synthesis that shows individualized practice. The present systematic review and meta-analysis evaluate the effects of TCM interventions guided by syndrome differentiation on sleep quality and blood pressure control in adults with both conditions. The analysis examines whether pattern-based interventions produce measurable improvements in sleep and blood pressure compared with non-differentiated TCM approaches or conventional care. The findings aim to inform clinical decision-making within integrated management models that address sleep disturbance and blood pressure regulation simultaneously.

## Methodology

### Research question and PICOS framework

In adults with hypertension and comorbid insomnia, does TCM based on syndrome differentiation improve sleep quality and blood pressure control compared with conventional treatment, placebo, sham intervention, or no treatment? The **Population (P)** for this meta-analysis includes adults aged 18 years or older who are diagnosed with primary hypertension and insomnia, as defined by the established medical diagnostic criteria in the included studies. all genders were eligible, with no restriction by ethnicity or geographic region, and participants were included from randomized controlled trails (RCTs) only to align with the eligibility criteria. **Intervention (I)** includes TCM therapies guided by syndrome differentiation, with treatment selection based on heart-kidney disharmony, liver yang hyperactivity, qi stagnation, qi deficiency, blood stasis, or mixed systems. The interventions included pattern-based TCM prescriptions, acupuncture, moxibustion, or combined TCM methods. The **Comparator (C)** included conventional Western medical care using antihypertensive drugs with sedative or hypnotic agents, placebo, sham TCM interventions, or no treatment, including standard care with or without non-differentiated TCM. The **Outcomes (O)** include sleep quality assessed using validated instruments like the Pittsburgh Sleep Quality Index (PSQI) and Insomnia Severity Index (ISI), and cardiovascular outcomes assessed using systolic and diastolic blood pressure, 24-hour ambulatory blood pressure, or predefined response measures, with secondary outcomes that include TCM syndrome scores, quality of life assessed using SF 36 or WHOQOL, and adverse events. The Study design (S) included randomized controlled trials (RCTs) for quantitative and qualitative synthesis when peer-reviewed full-text reports provided extractable data.

### Search strategy and study identification

**Figure 1:**
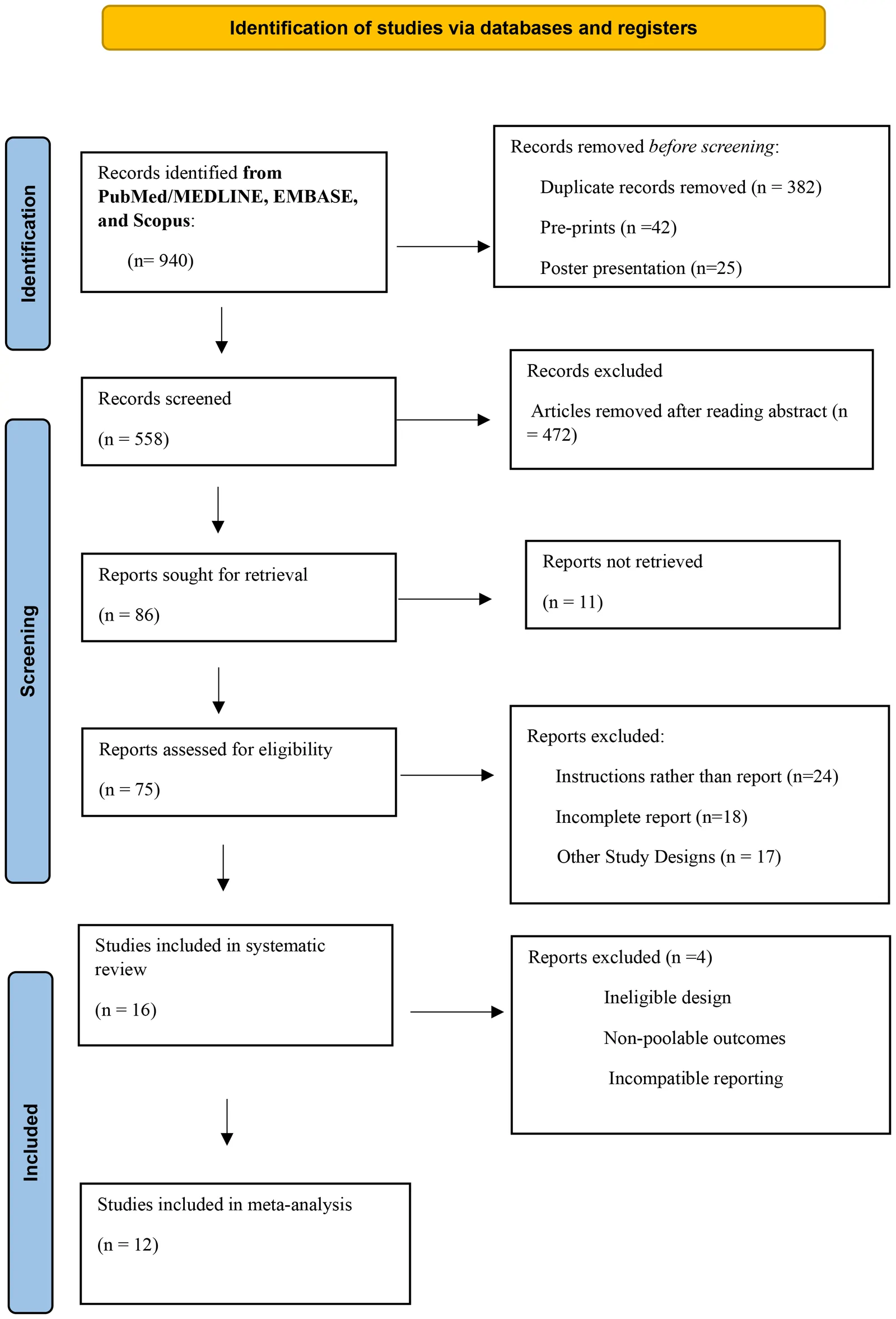
PRISMA flowchart for the review 2020.

The review protocol followed the Preferred Reporting Items for Systematic Reviews and Meta-Analyses (PRISMA) 2020 guidelines (**Figure 1**) (31). A comprehensive literature search was conducted in the multiple electronic databases like PubMed/MEDLINE, EMBASE, Scopus from 2013 to 2025. The search strategy combined controlled vocabulary and free-text terms related to hypertension, insomnia, TCM, and syndrome differentiation. Key search terms included “hypertension”, “essential hypertension”, “primary hypertension” “ambulatory blood pressure monitoring”, “insomnia”, “sleep disorder”, “sleep disturbance”, “sleep quality”, “Traditional Chinese Medicine”, “medicine, Chinese traditional”, “TCM”, “Chinese herbal medicine”, “herbal medicine”, “phytotherapy”, “syndrome differentiation”, “pattern differentiation”, “zheng”, “acupuncture”, “acupuncture therapy”, “herbal formula”, “circadian rhythm”, “autonomic nervous system”, “sympathetic nervous system”, “hypothalamic pituitary adrenal axis”, “quality of life”, and “randomized controlled trial”. Boolean operators “AND” and “OR” were applied appropriately. Reference lists of included articles were manually screened to identify additional eligible studies.

### Eligibility criteria

Studies enrolling adults with hypertension, insomnia, or both and receiving TCM interventions were included. Eligible studies reported quantitative sleep, blood pressure, or cardiovascular outcomes and applied recognized diagnostic criteria or clearly defined clinical diagnoses for hypertension or insomnia. Hypertension was defined as clinic blood pressure ≥140/90 mmHg on at least two occasions, daytime ambulatory blood pressure with SBP ≥135 mmHg and/or DBP ≥85 mmHg, 24-hour ambulatory blood pressure with SBP ≥130 mmHg and/or DBP ≥80 mmHg, or a guideline-based diagnosis with documented antihypertensive treatment. Insomnia was defined using DSM-IV, DSM-5, ICSD-3, or CCMD-3 criteria, PSQI scores >5 or >7, ISI scores ≥8 or ≥10, or clinical criteria comprising sleep onset latency >30 minutes, wake after sleep onset >30 minutes, total sleep time <6 hours, and daytime impairment occurring at least three nights per week for three months. Participants with hypertension, insomnia, or both conditions were eligible. Subgroup analyses compared treatment effects between comorbid and single-condition populations. Only randomized controlled trials that were published as peer-reviewed full-text articles were included in the review. Studies involving children, secondary hypertension, insomnia associated with major psychiatric or neurological disorders, animal or laboratory research, narrative reviews, editorials, conference abstracts lacking full text, and studies with non-extractable data were excluded.

### Study selection and data extraction

Two reviewers (Y. Fen and C. Xu) independently screened titles and abstracts. Full-text articles that met the initial selection criteria were independently assessed for eligibility. Disagreements were resolved through discussion. A third reviewer (Y. Ma) made the final decision when consensus was not reached. A total of 940 records were identified through database searches. Title and abstract screening, followed by full-text assessment, resulted in 16 eligible studies. Four studies were excluded from the quantitative synthesis due to design or reporting limitations. Twelve RCTs were included in the meta-analysis (**Figure 1**). The data included study design, sample size, participant characteristics, diagnostic criteria, syndrome classification, intervention and comparator details, treatment duration, outcome measures, adverse events, randomization methods, blinding procedures, and follow-up data.

### Statistical analysis and risk of bias assessment

The continuous outcomes were analyzed using mean differences or standardized mean differences (SMDs) with 95% confidence intervals (CIs), while dichotomous outcomes were analyzed using risk ratios. Statistical heterogeneity was assessed with the I² statistic and chi-square test. Random-effects models were used for moderate or high heterogeneity, whereas fixed-effect models were used for low heterogeneity. Subgroup analyses examined TCM syndrome categories, intervention types, and study features when sufficient data were available. Risk of bias in randomized controlled trials was assessed independently by two reviewers using the Cochrane Risk of Bias 2.0 tool (32), which included randomization, deviations from intended interventions, missing outcome data, outcome measurement, and selective reporting. Study quality was evaluated using the JADAD scale (33), with categories such as selection, comparability, and outcome domains. Publication bias was assessed visually using funnel plots of effect estimates against standard errors for outcomes with ≥10 studies; Egger’s linear regression test was not performed due to limited statistical power with fewer than 10 studies per outcome (34).

## Results

### Study characteristics and intervention features

The included trials (**Table 1**) differed in study design, population characteristics, intervention protocols, and syndrome classification. Sample sizes ranged from 60 to 662 participants. Treatment duration varied from 7 to 180 days, and follow-up extended to 12 months in one quasi-experimental study (20, 39–41, 43). Six of the 16 included studies enrolled participants with both hypertension and insomnia, five evaluated insomnia, and five evaluated hypertension. Study selection enabled assessment of syndrome-differentiated TCM interventions across different patient groups. Eligibility criteria, outcome measures, and intervention protocols varied between trials. Only a small number of randomized studies confirmed both conditions and applied predefined syndrome classification before enrolment (20, 38, 43). Interventions included acupuncture and herbal therapies, while comparators comprised sham procedures, antihypertensive medications, and usual care (37, 46).

**Table 1:** Characteristics of included studies evaluating syndrome-differentiated TCM for hypertension with insomnia.

Author, year	Country	Study design	Randomization method	Trial registration	Blinding strategy	Population (N)	Hypertension diagnostic criteria	Insomnia diagnostic criteria	TCM syndrome differentiation method	Intervention (Modality, formula/acupoints, duration)	Comparator	Primary outcomes reported
Guo J et al. (2013) (35)	China/Beijing Hospital of Traditional Chinese Medicine	Double-dummy, single-blind, randomized, placebo-controlled RCT	Computer-generated random sequence with block size = 6, 1:1:1 allocation	ISRCTN12585433	Double-dummy, single-blind (participants and outcome assessors blinded; acupuncturists not blinded)	180 (60 I/120	Not reported	Primary insomnia per DSM-IV-TR; duration ≥4 weeks	Not reported (fixed acupoint protocol used for all)	Verum acupuncture at DU24, EX-HN1, DU20, SP6, HT7; 3×/week × 6 weeks + placebo tablet	Estazolam + sham acupuncture or sham acupuncture + placebo tablet	PSQI, ESS, SF-36
Zheng H et al. (2019) (36)	China/11 hospitals (Sichuan, Hunan, Guizhou, Yunnan, Shaanxi)	Parallel RCT, assessor-blinded	Computer-generated random sequence in 1:1:1:1 ratio	NCT01617035	Single-blind (outcome assessors blinded; participants not blinded due to nature of acupuncture)	428 randomized (415 ITT)	Mild (Stage I) essential hypertension: SBP 140–159 mmHg and/or DBP 90–99 mmHg	Not reported	Not reported (no mention of TCM syndrome differentiation; used fixed acupuncture protocols: “affected meridian” or “non-affected meridian”)	Acupuncture (two active protocols pooled): 18 sessions over 6 weeks (3×/week)	Sham acupuncture + waiting-list control	Change in 24-h ambulatory SBP at week 6
Zhang DY et al. (2020) (37)	China/Single center (Shanghai Institute of Hypertension)	Parallel RCT, double-blind, placebo-controlled	Stratified randomization by center, sex, and TCM syndrome (LYHS vs. non-LYHS) using computer-generated sequence	NCT03739618	Double-blind (participants and investigators)	251 (126 I/125 C)	Masked hypertension: clinic BP <140/90 mmHg + daytime ambulatory SBP 135–150 mmHg or DBP 85–95 mmHg	Not reported	TCM syndromes categorized (e.g., liver-yang hyperactivity syndrome [LYHS]), but treatment was not individualized—all received same GUG formula regardless of pattern	Gastrodia-uncaria granules (GUG), 5 g twice daily for 2 weeks, then uptitrated to 10 g twice daily for 2 more weeks (total 4 weeks)	Matching placebo granules, identical regimen	Change in daytime ambulatory SBP at 4 weeks
Siu PM et al. (2021) (14)	Hong Kong/Single research unit	Randomized, 3-arm, parallel-group, assessor-masked clinical trial	Automated permuted block algorithm (block size = 30)	NCT02260843	Assessor-masked (outcome assessors blinded; participants/instructors not blinded due to intervention nature)	320 (105 Tai Chi/105 Exercise/110 Control)	Not reported	Chronic insomnia per DSM-5 criteria	Not applicable (Tai Chi is a mind-body exercise, not a TCM syndrome-differentiated therapy)	Yang-style 24-form Tai Chi, 3 sessions/week × 12 weeks	Conventional exercise (brisk walking + strength training) or passive control (usual care)	Actigraphy-assessed sleep efficiency, wake time after sleep onset, number of awakenings
Wang C et al. (2021) (38)	China/Yueyang Hospital of Integrated Traditional Chinese and Western Medicine, Shanghai	Randomized, single-blind, parallel, sham-controlled RCT	Computer-generated random sequence (SPSS 25.0, seed = 20190520), 1:1 allocation	ChiCTR1900023787	Single-blind (participants and outcome assessors blinded; acupuncturist not blinded)	82 (41 I/41 C)	Not reported	Chronic insomnia per ICSD-3 + PSQI >5	TCM syndrome of “dysregulated coordination between heart and kidney” required for inclusion	Acupuncture at bilateral HT7 (Shenmen) and KI7 (Fuliu), 10 sessions over 3 weeks (3×/week, 20 min/session)	Sham acupuncture (superficial needling 2 cun lateral to true points), same regimen	PSQI, ISI
Fang Y et al. (2021) (39)	China/Beijing University of Chinese Medicine	Non-randomized controlled trial (quasi-experimental)	Not reported	Not reported	Not reported	110 (60 I/50 C)	Chinese Guidelines for the Prevention and Treatment of Hypertension (2010 Revision)	Not reported	Body constitution identified via Constitution in Chinese Medicine Questionnaire (CCMQ); treatment tailored to 9 constitution types (e.g., Yin-deficiency → Fructus Lycii; Blood-stasis → Salvia miltiorrhiza) + acupuncture/moxibustion	Body constitution-identified TCM syndrome differentiation treatment (herbal dietary therapy + acupuncture at Shenmen, Erjian, and adjunct points) for 12 months	Conventional Western medical care (antihypertensives, hypoglycemics, lifestyle advice)	Changes in SBP, DBP, fasting glucose, 2hPG, SF-36 quality of life
Xia N et al. (2022) (20)	China/single-center tertiary hospital	Double-blind randomized placebo-controlled trial	Block randomization with 1:1 allocation; sequence generated by independent trial administrator	ChiCTR1800019239	Double-blind (participants, clinicians, assessors, statisticians)	128 randomized (64 JTW, 64 placebo); 106 completed	NA	CCMD-3; PSQI ≥7	Disharmony of heart and kidney diagnosed per Internal Medicine of TCM; DHKSS ≥9	TCM; Jiaotaiwan granules 2 g twice daily for 7 days	Placebo granules	PSQI total score
Lai X et al. (2022) (26)	China/17 centers nationwide	Multicenter, randomized, double-blind, double-dummy, active-controlled, noninferiority RCT	Computer-generated permuted blocks (block size = 4), 1:1 allocation	ChiCTR-IPR-16008108	Double-blind (participants, investigators, outcome assessors, statisticians)	628 (314 I/314 C)	Mild (Grade 1) essential hypertension per 2010 Chinese Guidelines: SBP 140–159 mmHg and/or DBP 90–99 mmHg	Not formally diagnosed; sleep quality assessed via Pittsburgh Sleep Quality Index (PSQI) as a secondary outcome	Not reported (fixed SXC formula given to all; no individualization by TCM pattern)	Songling Xuemaikang capsule (SXC): 0.5 g × 3 capsules, tid × 8 weeks (total 4.5 g/day)	Losartan 50 mg once daily × 8 weeks	Change in sitting diastolic BP (SiDBP) at 8 weeks
Xu X et al. (2023) (40)	China/Multicenter (Xiyuan Hospital, First Affiliated Hospital of Guangxi University of Chinese Medicine, Jiangxi Provincial People’s Hospital)	Multicenter, randomized, double-blind, placebo-controlled trial	Block randomization, 1:1 ratio	ChiCTR1900021699	Double-blind (participants, investigators, outcome assessors); identical-appearing capsules	60 randomized; 58 in FAS (29 I/29 C); 51 completed	Grade 1 essential hypertension (SBP 140–159 mmHg and/or DBP 90–99 mmHg) with low-to-moderate cardiovascular risk	Not reported (insomnia not assessed)	Not applicable (fixed Quanduzhong capsule given to all; no individualized TCM pattern diagnosis)	Quanduzhong capsules (3 capsules twice daily, containing Eucommia ulmoides extract), for 4 weeks	Matching placebo capsules, identical regimen	Primary: 24-h ambulatory SBP/DBP, office SBP/DBP Secondary: BP variability (CV), home BP, WHOQOL-BREF, hypertension symptom scores, homocysteine, renin-angiotensin markers
Xie J et al. (2024) (23)	China/Yueyang Hospital of Integrated Traditional Chinese and Western Medicine, Shanghai University of TCM	Randomized, double-blind, placebo-controlled RCT	Computer-generated random sequence	ChiCTR2000035092	Double-blind (participants and investigators)	216 (108 I/108 C)	Grades 1 or 2 essential hypertension (per Chinese guidelines, implied)	Not reported	Participants selected based on TCM syndromes: blood stasis, yang hyperactivity, or phlegm	Huoxue Qianyang Qutan Recipe (HQQR) granules + conventional antihypertensive treatment, 12 weeks	Placebo granules + conventional antihypertensive treatment, 12 weeks	Clinic systolic and diastolic blood pressure
Xiong ZY et al. (2024) (17)	China/Not specified (likely tertiary hospital)	Randomized, placebo-controlled, triple-blinded RCT	Computer-generated random sequence	NCT01613183	Triple-blinded (participants, clinicians, outcome assessors)	116 (96 I/20 C)	Not reported	Moderate to severe primary insomnia (age 18–65); diagnostic criteria not specified in abstract (likely DSM-IV or CCMD-3)	Individualized herbal prescriptions by three independent CM clinicians based on syndrome differentiation (most common: blood deficiency and liver stagnation)	Individualized Chinese herbal medicine decoctions, tailored to each patient’s syndrome, 4 weeks	Matching placebo, 4 weeks	Change in total sleep time (TST)
Xin Q et al. (2024) (41)	China	Double-blind, randomized, parallel-group, placebo-controlled RCT	Computer-generated random sequence, 1:1 allocation	NCT05764798	Double-blind (participants, investigators, outcome assessors)	60 (30 I/30 C)	Not reported	Insomnia Disorder with Depressive Symptoms (IDDS); diagnosis based on clinical criteria + ISI ≥ 15, PHQ-9 ≥ 10	Not reported (Shugan Jieyu used as fixed formula, not individualized by TCM pattern)	Shugan Jieyu capsules (0.72 g twice daily) + zolpidem (10 mg once daily), 8 weeks	Placebo capsules + zolpidem (10 mg once daily), 8 weeks	Change in Insomnia Severity Index (ISI) score at week 8
Zhao J et al. (2025) (42)	China/Affiliated Hospital of Gansu University of Chinese Medicine	Randomized controlled trial	Not specified	Not reported	Not reported (open-label)	90 (45 I/45 C)	Not reported	PSQI >7 + clinical insomnia symptoms ≥3×/week; aligned with Brazilian Sleep Association 2023 guidelines	TCM syndrome of Qi deficiency and blood stasis confirmed per “Diagnostic and Therapeutic Evaluation Criteria for Stroke (Trial)”	Self-made Jianpi Huoxue Jieyu formula (decoction, 300 mL twice daily) for 4 weeks, added to standard post-stroke care	Standard post-stroke care only (antihypertensives, antiplatelets, lipid/glucose control, diet)	PSQI, TCM symptom score, NIHSS
Wang F et al. (2025) (43)	China/Multicenter (19 sites)	Multicenter, randomized, double-blind, placebo-controlled trial	1:1 randomization (method not specified beyond ratio)	ChiCTR1900022470	Double-blind (participants and investigators)	662 enrolled; 608 completed (306 I/302 C)	Grade 1 hypertension (SBP 140–159 mmHg and/or DBP 90–99 mmHg) per Chinese guidelines	Not formally diagnosed; insomnia assessed as a symptom within CM syndrome	TCM syndrome of blood deficiency and Gan (Liver)-yang hyperactivity required for inclusion; diagnosis based on CM pattern differentiation	Yangxue Qingnao Pills (YXQNP) + amlodipine besylate, oral, for 180 days	YXQNP placebo + amlodipine besylate, identical regimen	Change in BP variability, CM syndrome scores, symptom improvement (dizziness, headache, insomnia, waist soreness), target organ markers (baPWV, ABI, ACR), adverse events
Xiang Y et al. (2025 ) (44)	China/Multicenter (9 hospitals across China)	Randomized, double-blind, positive-controlled, multicenter RCT	Computer-generated random sequence with block size = 4, stratified by center, 1:1 allocation	ChiCTR1800017827	Double-blind (identical-appearing capsules/tablets; participants, clinicians, data managers masked)	198 randomized; 183 completed (92 I/91 C)	Not reported (hypertension not assessed or required)	Insomnia assessed as a somatic complaint; no formal insomnia diagnosis (e.g., DSM/ICSD); PSQI used as secondary outcome	TCM syndrome of “liver-qi stagnation and spleen deficiency”	Shugan Jieyu capsule (0.72 g twice daily) + St. John’s wort placebo, oral, for 8 weeks	St. John’s wort tablet (0.56 g twice daily) + Shugan Jieyu placebo, identical regimen	Primary: change in HDRS-17 score Secondary: PSQI, PHQ-15 (somatic symptoms), HAMA, Q-LES-Q-SF, adverse events
Pang HY et al. (2025) (45)	China/Xiyuan Hospital, China Academy of Chinese Medical Sciences	Randomized controlled trial	Random number table, 1:1 allocation	ChiCTR2200063884	Not blinded (open-label design)	94 randomized; 84 completed (42 I/42 C); FAS = 47 per group	Not reported (hypertension not assessed or required)	Insomnia Disorder (ID) per clinical diagnosis (criteria not specified); ISI and PSQQI used as measures	Not stated; Jianpi Jieyu Decoction (JJD) implies treatment for “spleen deficiency and liver stagnation” pattern, but no formal syndrome assessment method described	Yu Melody Relaxation Training (YMRT) + oral Jianpi Jieyu Decoction (JJD), for 4 weeks + 2-week follow-up	Oral JJD alone, same duration	Primary: change in ISI score at week 4 Secondary: ISI response rate, PSQI, PHQ-9, GAD-7 at weeks 1–6, adverse events

RCT, randomized controlled trial; ITT, intention-to-treat population; FAS, full analysis set; SBP, systolic blood pressure; DBP, diastolic blood pressure; BP, blood pressure; PSQI, Pittsburgh Sleep Quality Index; ISI, Insomnia Severity Index; ESS, Epworth Sleepiness Scale; SF-36, Short Form–36 Health Survey; WHOQOL-BREF, World Health Organization Quality of Life–BREF; DSM-IV-TR, Diagnostic and Statistical Manual of Mental Disorders, Fourth Edition, Text Revision; DSM-5, Diagnostic and Statistical Manual of Mental Disorders, Fifth Edition; ICSD-3, International Classification of Sleep Disorders, Third Edition; CCMD-3, Chinese Classification of Mental Disorders, Third Edition; LYHS, liver-yang hyperactivity syndrome; DHKSS, Disharmony of Heart–Kidney Syndrome Score; CCMQ, Constitution in Chinese Medicine Questionnaire; CV, coefficient of variation; baPWV, brachial–ankle pulse wave velocity; ABI, ankle–brachial index; ACR, albumin-to-creatinine ratio; HDRS-17, 17-item Hamilton Depression Rating Scale; HAMA, Hamilton Anxiety Rating Scale; PHQ-9, Patient Health Questionnaire-9; PHQ-15, Patient Health Questionnaire-15; Q-LES-Q-SF, Quality of Life Enjoyment and Satisfaction Questionnaire–Short Form; TCM, Traditional Chinese Medicine.

### Sleep and blood pressure outcomes

The RCTs presented in **Table 2** showed variation in sleep and blood pressure outcomes. Baseline PSQI scores ranged from 11 to 15. Studies that classified patients by syndrome before treatment showed larger improvements in PSQI scores, with reductions ranging from 1.64 to 3.5 points (20, 38, 45). The trials that used fixed formulas or adjunctive treatment designs reported smaller changes or no statistically significant differences between groups (41, 44). Blood pressure outcomes were reported in fewer studies. Blood pressure outcomes were reported less frequently than sleep outcomes. Systolic blood pressure reductions ranged from 2.5 to 6.2 mmHg, whereas changes in diastolic blood pressure were generally small and often non-significant. One multicentre trial reported reduced blood pressure variability. Adverse events were mild, and follow-up duration ranged from 1 to 26 weeks (23, 37, 43).

**Table 2:** Quantitative effects of syndrome-differentiated TCM on sleep quality and blood pressure in patients with hypertension and insomnia (RCTs).

Author, year	Baseline PSQI (I/C)	Post-treatment PSQI (I/C)	ΔPSQI (MD†, 95% CI)	Baseline SBP (mmHg) (I/C)	Post-treatment SBP (mmHg) (I/C)	ΔSBP (MD†, 95% CI)	Baseline DBP (mmHg) (I/C)	Post-treatment DBP (mmHg) (I/C)	ΔDBP (MD†, 95% CI)	Adverse events (I/C)	Follow-up (weeks)
Guo J et al. (2013) (35)	11.5 ± 2.0/12.0 ± 2.0	6.8 ±?/9.5 ±?	−2.7 [95% CI not reported]	Not reported	Not reported	Not reported	Not reported	Not reported	Not reported	25/47	6
Zheng H et al. (2019) (36)	Not reported	Not reported	Not reported	147.8 ± 5.8/147.5 ± 5.4	140.6 ±?/143.4 ±?	2.9 [−0.2, 6.0] (vs. waiting-list at week 6)	89.8 ± 7.7/89.5 ± 7.3	86.1 ±?/86.2 ±?	0.4 [−2.2, 3.1] (vs. waiting-list at week 6)	5/4 (active vs. sham acupuncture)	6 (treatment); 12 (total follow-up)
Zhang DY et al. (2020) (37)	Not reported	Not reported	Not reported	135.6 ± 6.8/134.4 ± 7.1 (daytime ambulatory)	130.2 ± 9.1/131.5 ± 9.8	2.52 [0.32, 4.73]	89.8 ± 4.7/89.1 ± 4.5 (daytime ambulatory)	86.4 ± 6.4/87.5 ± 6.9	1.79 [0.41, 3.17]	0/1	4
Siu PM et al. (2021) (14)	11.3 ± 2.9/11.4 ± 3.2	7.9 ± 3.8/10.9 ± 3.5	−2.9 [−3.6, −2.1]	Not reported	Not reported	Not reported	Not reported	Not reported	Not reported	0/0	12
Wang C et al. (2021) (38)	14.78 ± 2.71/14.61 ± 3.11	9.74 ± 3.08/11.69 ± 3.65	−1.95 [−3.42, −0.48]	Not reported	Not reported	Not reported	Not reported	Not reported	Not reported	0/1	3 (treatment); 4 (follow-up)
Fang Y et al. (2021) (39)	Not reported	Not reported	Not reported	Not reported	Significantly lower in I vs C at 12 months (P<0.05)	Not reported	Not reported	Significantly lower in I vs C at 12 months (P<0.05)	Not reported	Not reported	52 (12 months)
Xia N et al. (2022) (20)	12.50 ± 2.97/12.03 ± 2.82	9.34 ± 3.58/10.98 ± 3.07	−1.64 [−2.81, −0.47]	Not reported	Not reported	Not reported	Not reported	Not reported	Not reported	0/0	1
Lai X et al. (2022) (26)	Not reported	Not reported	No significant difference (P > 0.05)	145 ±?/145 ±?	134.5 ± 10.3/134.4 ± 10.3	−0.1 [−1.8, 1.6]	92 ±?/92 ±?	84.1 ± 8.0/83.9 ± 7.9	−0.24 [−1.51, 1.03]	Similar incidence (exact numbers not specified)	8
Xu X et al. (2023) (40)	Not reported	Not reported	Not reported	144.69 ± 7.31/147.10 ± 7.55	137.07 ± 8.92/143.55 ± 9.73	−4.07 [95% CI not reported; p = 0.03]	90.31 ± 6.52/91.90 ± 5.95	85.66 ± 8.20/91.52 ± 8.75	−4.28 [95% CI not reported; p = 0.01]	Mild AEs: 12/29 (I) vs. 10/29 (C); no significant difference (p = 0.66)	4
Xie J et al. (2024) (23)	Not reported	Not reported	Not reported	Not reported	130.06 ± 8.50/136.24 ± 7.63	−6.18 [95% CI not reported]	Not reported	80.46 ± 6.05/84.34 ± 8.72	−3.88 [95% CI not reported]	0/0	12
Xiong ZY et al. (2024) (17)	Not reported	Not reported	−2.0 [−3.2, −0.8	Not reported	Not reported	Not reported	Not reported	Not reported	Not reported	Mild, unrelated to treatment (exact numbers not specified)	4 (treatment) + 4 (follow-up)
Xin Q et al. (2024) (41)	11.70 ± 3.19/12.31 ± 3.33	8.29 ±?/9.15 ±?	−0.25 [−1.90, 1.40]	Not reported	Not reported	Not reported	Not reported	Not reported	Not reported	23/25	8
Zhao J et al. (2025) (42)	13.47 ± 0.87/13.68 ± 0.95	6.06 ± 0.64/9.00 ± 0.57	−2.94 [95% CI not reported]	Not reported	Not reported	Not reported	Not reported	Not reported	Not reported	1/0	4
Wang F et al. (2025) (43)	Not reported	Not reported	Not reported	Not reported	Not reported	Not reported (but BP variability ↓ significantly in I vs C)	Not reported	Not reported	Not reported	No serious AEs; minor AEs (nausea, vomiting, rash, itching, diarrhea) recorded but exact numbers not reported	26 (180 days)
Xiang Y et al. (2025 ) (44)	12.80 ± 3.55/12.25 ± 3.15	6.91 ± 3.60/6.98 ± 3.43	−0.47 [95% CI not reported; p = 0.415]	Not reported	Not reported	Not reported	Not reported	Not reported	Not reported	8/99 (8.08%)/6/99 (6.06%); no serious AEs (p = 0.774)	8
Pang HY et al. (2025) (45)	Not reported	Week 4: Not reported Week 6: Significantly lower in I vs C	−3.5 [95% CI: −5.21 to −1.85] at week 6	Not reported	Not reported	Not reported	Not reported	Not reported	Not reported	4/47 (8.51%)/2/47 (4.26%); p > 0.05	6 (4 weeks treatment + 2 weeks follow-up)

PSQI, Pittsburgh Sleep Quality Index; SBP, systolic blood pressure; DBP, diastolic blood pressure; BP, blood pressure; MD, mean difference; CI, confidence interval; AEs, adverse events; NA, not assessed or not applicable;?, data not reported in the original study; daytime ambulatory BP, blood pressure measured by daytime ambulatory monitoring; BP variability, variability indices reported by original authors.

**Table 3** summarizes syndrome-specific sleep and blood pressure outcomes reported in studies of TCM interventions. Sleep outcomes were most frequently reported in deficiency and disharmony syndromes. Acupuncture for dysregulated heart–kidney coordination reduced PSQI scores by 1.95 points (38), while Jiaotaiwan granules reduced PSQI scores by 1.64 points in patients with heart–kidney disharmony (20). Individualized herbal prescriptions for blood deficiency with liver stagnation produced a PSQI-equivalent reduction of 2.0 points (17), and Jianpi Huoxue Jieyu therapy reduced PSQI scores by 2.94 points in patients with qi deficiency and blood stasis (42). Blood pressure outcomes were reported primarily in liver-yang hyperactivity and blood stasis syndromes. Gastrodia–Uncaria granules reduced daytime ambulatory systolic blood pressure by 2.52 mmHg (37), and the Huoxue Qianyang Qutan Recipe, combined with antihypertensives, reduced clinic systolic blood pressure by 6.18 mmHg (23). A multicentre trial reported improvements in insomnia and blood pressure variability within a single syndrome-defined population (43).

**Table 3:** Pattern-specific effects of syndrome-differentiated TCM on sleep quality and blood pressure in adults with hypertension and/or insomnia.

TCM syndrome pattern	Study (Author, Year)	Intervention	Sleep outcome (Instrument)	Effect on sleep	Blood pressure outcome	Effect on blood pressure
Dysregulated coordination between heart and kidney	Wang C et al. (2021) (38)	Acupuncture (HT7, KI7) [pattern required for enrollment]	PSQI, ISI	PSQI reduction by 1.95 (95% CI −3.42 to −0.48)	Not reported	NA
Blood deficiency with liver-yang hyperactivity	Wang F et al. (2025) (43)	Yangxue Qingnao Pills plus amlodipine [pattern required for enrollment]	Insomnia symptom score (CM scale)	Response rate increased by 34.05% (p<0.05)	Office BP variability (coefficient of variation)	Significant reduction (p<0.05)
Heart-kidney disharmony	Xia N et al. (2022) (20)	Jiaotaiwan granules [pattern required for enrollment]	PSQI	PSQI reduction by 1.64 (95% CI −2.81 to −0.47)	Not reported	NA
Qi deficiency with blood stasis	Zhao J et al. (2025) (42)	Jianpi Huoxue Jieyu formula [pattern required for enrollment]	PSQI	PSQI reduction by 2.94	Not reported	NA
Blood deficiency with liver stagnation	Xiong ZY et al. (2024) (17)	Individualized herbal decoctions [clinician-led individualized prescription]	Total sleep time (converted to PSQI equivalent)	PSQI-equivalent reduction by 2.0 (95% CI −3.2 to −0.8)	Not reported	NA
Liver-yang hyperactivity	Zhang DY et al. (2020) (37)	Gastrodia–Uncaria granules [pattern used for stratification; fixed intervention]	Not assessed	NA	Daytime ambulatory BP	SBP reduction by 2.52 mmHg
Blood stasis/yang hyperactivity/phlegm	Xie J et al. (2024) (23)	Huoxue Qianyang Qutan Recipe plus antihypertensives [pattern required for enrollment]	Not assessed	NA	Clinic BP	SBP reduction by 6.18 mmHg

PSQI, Pittsburgh Sleep Quality Index; ISI, Insomnia Severity Index; SBP, systolic blood pressure; BP, blood pressure; CI, confidence interval; NA, not assessed or not reported; CM scale, Chinese Medicine–based symptom scoring system; PSQI-equivalent, sleep outcome converted to PSQI-based metric by original authors; daytime ambulatory BP, blood pressure measured using ambulatory monitoring during daytime hours. Bracketed annotations indicate how TCM syndrome pattern was applied in each study: [pattern required for enrollment] = participants had to meet this syndrome criterion; [stratification] = pattern used for randomization stratification but intervention was fixed; [individualized] = treatment tailored per real-time syndrome assessment.

### Risk of bias and methodological quality assessment

**Figure 2:**
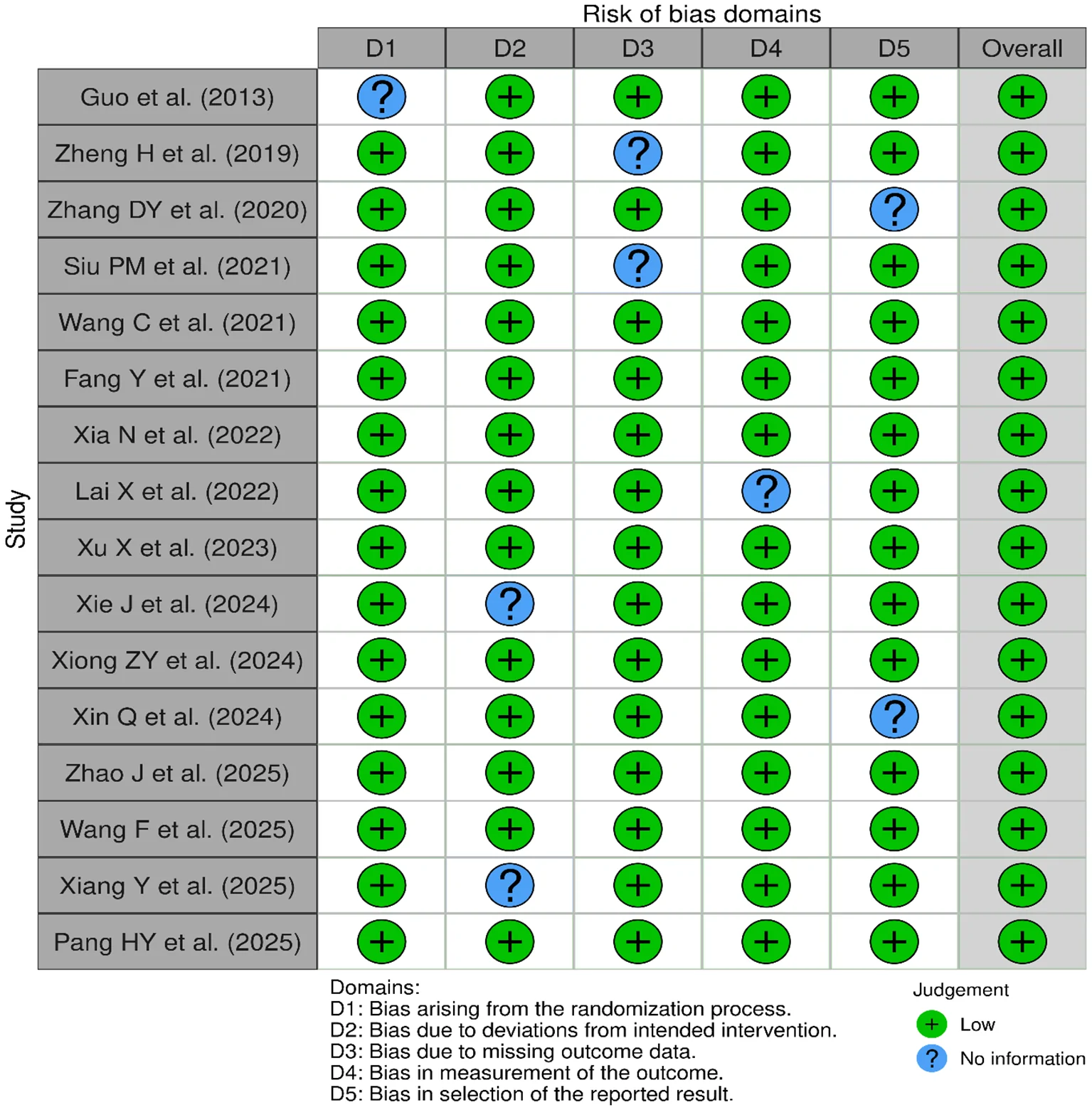
Risk of bias assessment of individual studies using the ROB2 tool. Green circle with plus symbol indicates low risk of bias; blue circle with question mark indicates some concerns due to insufficient or unclear information; no high-risk judgments were identified. D1, bias arising from the randomization process; D2, bias due to deviations from intended interventions; D3, bias due to missing outcome data; D4, bias in measurement of the outcome; D5, bias in selection of the reported result. Overall judgment reflects the highest risk level across domains for each study.

**Figure 2** presents the ROB2 assessment of the included randomized controlled trials. Most domains were rated as low risk. Randomization bias was identified in Guo J et al. (2013) (35) because allocation concealment was insufficiently described. Limited reporting of participant blinding, intervention adherence, or attrition handling contributed to bias related to deviations from intended interventions and missing outcome data in some studies (14, 23, 36, 44). Outcome measurement bias was identified in Lai X et al. (2022) (26) due to unclear assessor blinding for sleep outcomes. Reporting bias was identified in Zhang DY et al. (2020) (37) and Xin Q et al. (2024) (41) because prespecified analysis plans were unavailable. No study was rated as high risk in any ROB2 domain.

**Figure 3:**
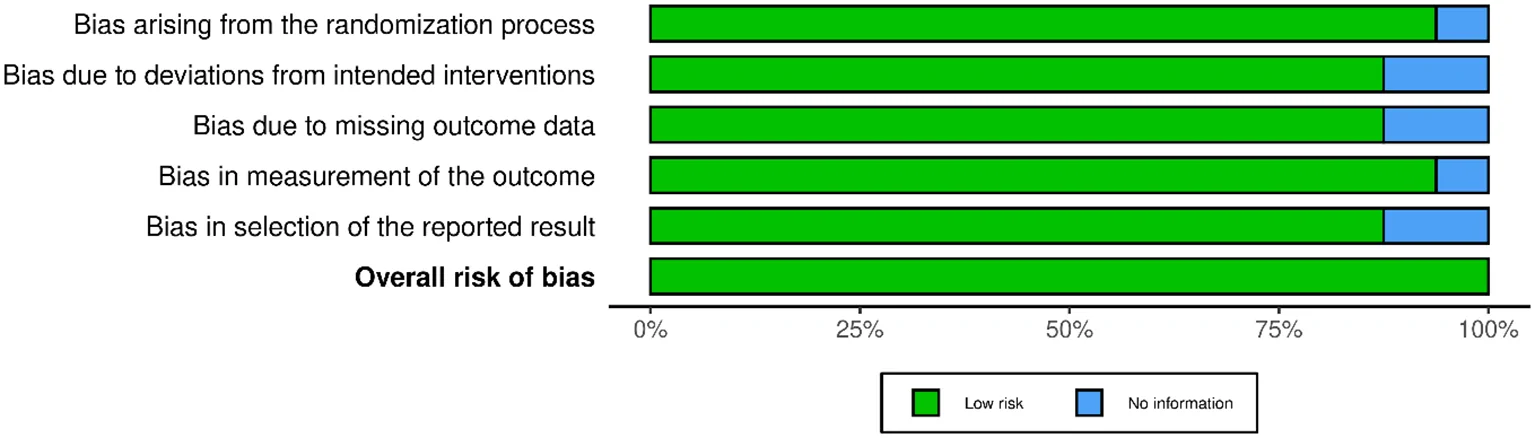
Risk of bias assessment overall studies with ROB2 tool. Green bars represent the proportion of studies judged as low risk of bias; blue bars represent the proportion of studies with some concerns due to insufficient or unclear information; no studies were rated as high risk of bias. Bias domains are defined as follows: bias arising from the randomization process; bias due to deviations from intended interventions; bias due to missing outcome data; bias in measurement of the outcome; bias in selection of the reported result. Overall risk of bias reflects the highest level of concern identified across domains for each study.

**Figure 3** shows the distribution of ROB2 ratings for the included randomized controlled trials. Low-risk ratings predominated in all domains. Most studies reported appropriate random sequence generation, but insufficient descriptions of allocation concealment introduced some uncertainty in the randomization domain. Deviations from intended interventions and missing outcome data were generally rated as low risk, with uncertainty arising from incomplete reporting of participant blinding, protocol adherence, attrition handling, or intention-to-treat analysis. Outcome measurement was largely supported by validated sleep questionnaires and standardized blood pressure assessment methods, although assessor blinding was not always specified. Reporting bias was infrequent and mainly related to the unavailability of prespecified analysis plans. No domain demonstrated a substantial proportion of high-risk ratings, indicating satisfactory methodological quality of the included randomized trials.

**Table 4** presents methodological quality scores of the included randomized controlled trials using the Jadad scale (33) (maximum score = 5). 12 studies scored 3, with reported randomization and withdrawals and no or unclear blinding (17, 20, 23, 35, 36, 38–40, 42, 43, 45). 3 studies scored 5 with reporting of randomization, double blinding, and withdrawals (14, 44, 46). One study scored 4 with reported randomization, partial blinding, and withdrawals (41). No study scored <3. All trials met the threshold for high quality (≥3). Differences in scores reflect the presence or absence of blinding components in the Jadad scoring criteria.

**Table 4:** Quality assessment for the randomized controlled trial (JADAD scale).

Author (year)	Randomization (Q1)		Blinding (Q2)		Withdrawals (Q3)	Total score	Quality
	Q1a	Q1b	Q2a	Q2b	Q3	(Max 5)	(≥3 = High)
Guo J et al. (2013) (35)	1	1	0	0	1	3	High
Zheng H et al. (2019) (36)	1	1	0	0	1	3	High
Zhang DY et al. (2020) (37)	1	1	0	0	1	3	High
Siu PM et al. (2021) (14)	1	1	1	1	1	5	High
Wang C et al. (2021) (38)	1	1	0	0	1	3	High
Fang Y et al. (2021) (39)	1	1	0	0	1	3	High
Xia N et al. (2022) (20)	1	1	0	0	1	3	High
Lai X et al. (2022) (26)	1	1	1	1	1	5	High
Xu X et al. (2023) (40)	1	1	0	0	1	3	High
Xie J et al. (2024) (23)	1	1	0	0	1	3	High
Xiong ZY et al. (2024) (17)	1	1	0	0	1	3	High
Xin Q et al. (2024) (41)	1	1	1	0	1	4	High
Zhao J et al. (2025) (42)	1	1	0	0	1	3	High
Wang F et al. (2025) (43)	1	1	0	0	1	3	High
Xiang Y et al. (2025) (44)	1	1	1	1	1	5	High
Pang HY et al. (2025) (45)	1	1	0	0	1	3	High

### Quantitative synthesis of primary outcomes

**Figure 4:**
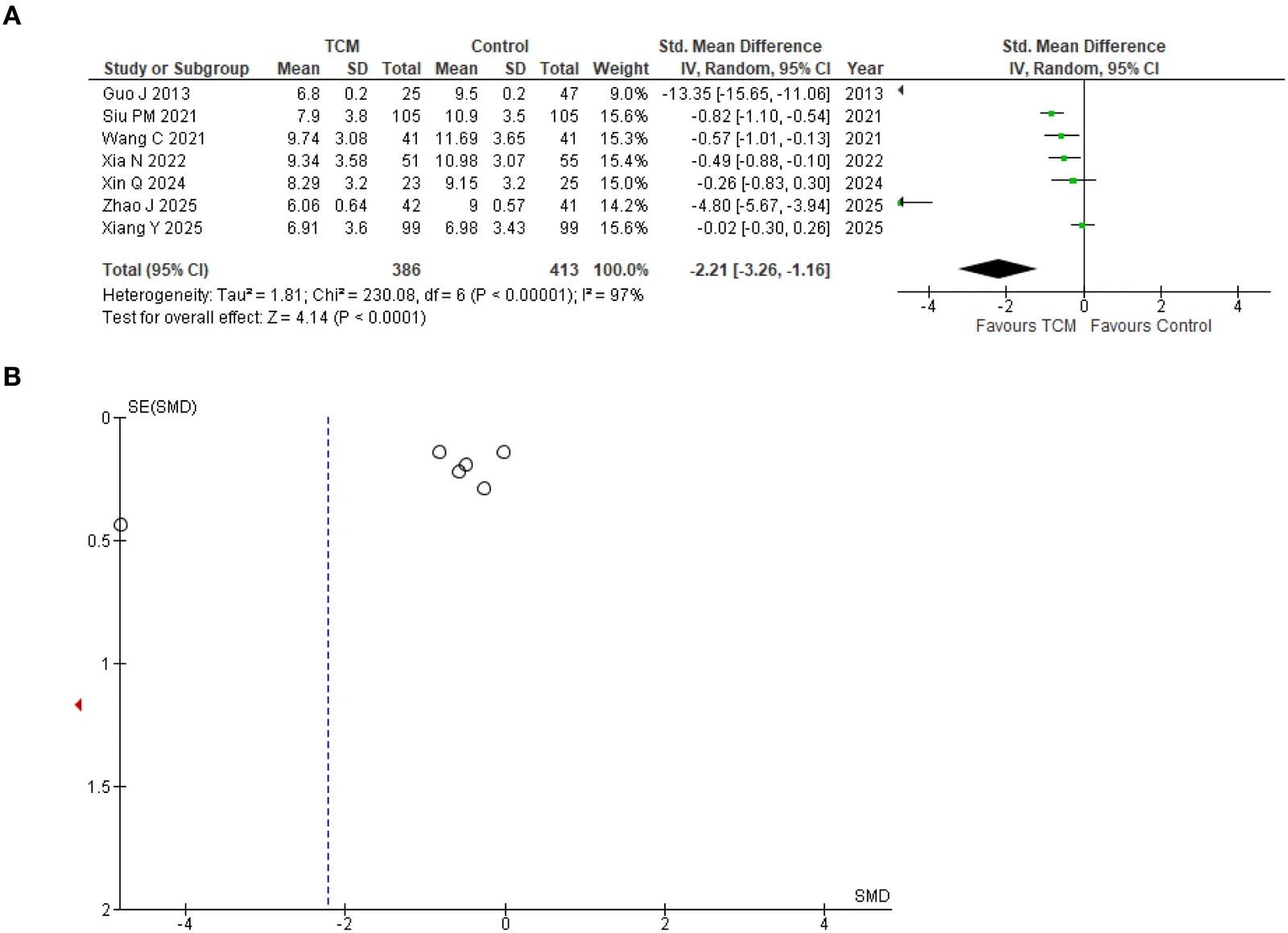
**(a)** Forest plot of pooled standardized mean differences in sleep quality (PSQI scores) comparing syndrome-differentiated TCM with control interventions in RCTs. **(b)** Funnel plot assessing publication bias for sleep quality outcomes (SMD) in included randomized controlled trials.

Four studies were excluded from the quantitative synthesis due to design and data limitations (**Figure 1**). Fang Y et al. (2021) (39) was non-randomized, reported outcomes without mean or dispersion values, used a prolonged follow-up, and applied combined interventions. Xiong ZY et al. (2024) (17) evaluated insomnia only using total sleep time, with no PSQI/ISI or blood pressure data. Wang F et al. (2025) (43) reported blood pressure variability and symptom scores without extractable SBP or DBP means. Pang HY et al. (2025) (45) did not assess hypertension, reported insomnia-only outcomes, and had deficiencies in harmonized time points and variance measures. **Figure 4a** presents PSQI outcomes from 7 RCTs, with 386 participants in the intervention group and 413 in the control group. Large reductions in PSQI scores were reported by some studies (35, 42), while others reported moderate but statistically significant improvements (14, 20, 38). Confidence intervals crossed the null value in a few studies (41, 44). Meta-analysis demonstrated a significant reduction in PSQI scores in favor of TCM interventions (SMD −2.21, 95% CI −3.26 to −1.16; Z = 4.14, p < 0.0001). Heterogeneity was high (I² = 97%). Subgroup analyses based on intervention modality, syndrome restriction status, and treatment duration showed significant between-group differences (Q-between p < 0.05). High heterogeneity persisted within subgroups (I² = 86–98%), indicating variation related to syndrome classification, intervention type, baseline insomnia severity, and treatment duration.

**Figure 5:**
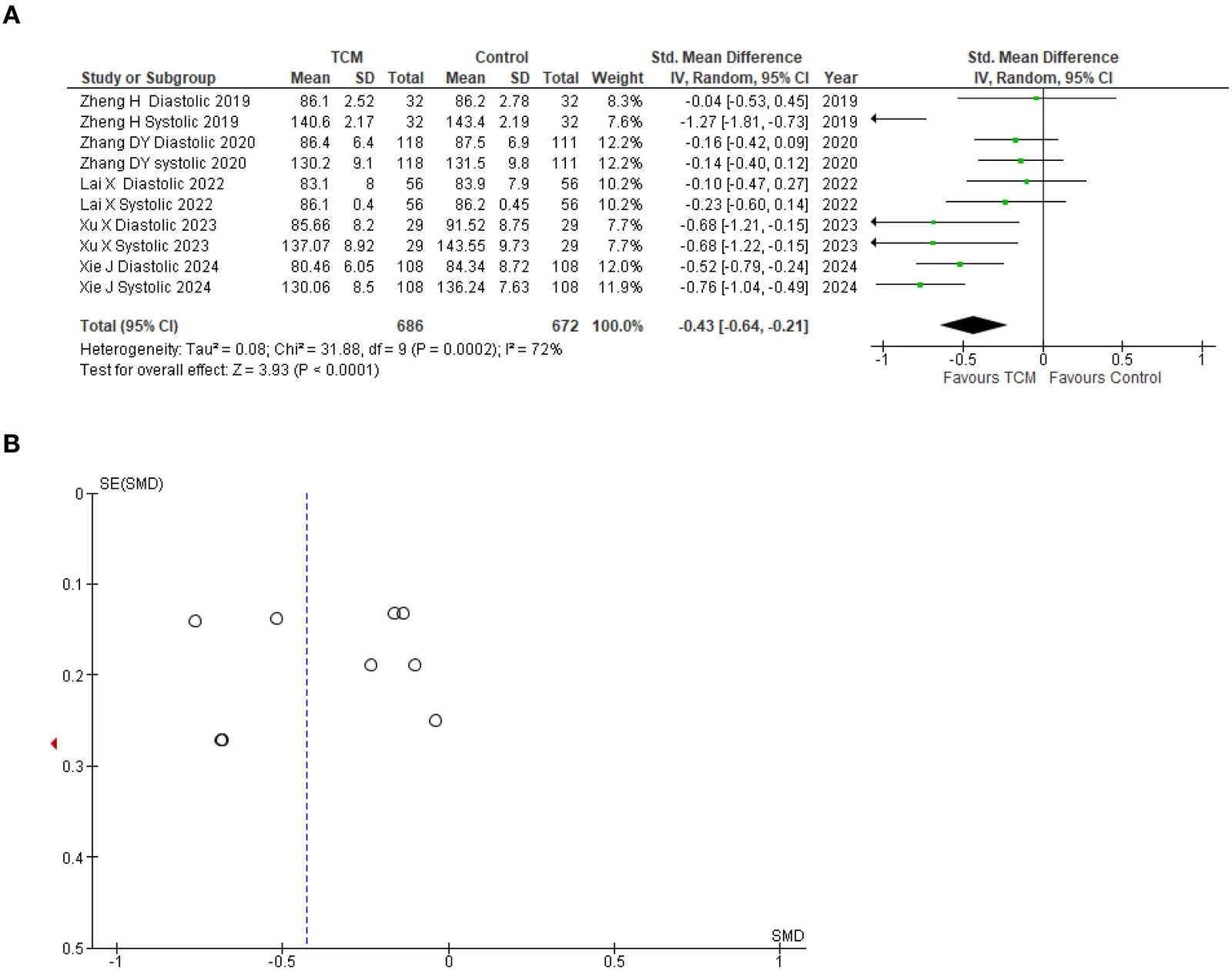
**(a)** Forest plot of pooled standardized mean differences in systolic and diastolic blood pressure comparing syndrome-differentiated TCM with control interventions in RCTs. **(b)** Funnel plot for assessment of publication bias in blood pressure outcomes (SMD) in the included randomized controlled trials.

**Figure 5a** presents blood pressure outcomes from 10 comparisons across 5 RCTs, including 686 participants in the intervention group and 672 in the control group. Several studies reported significant reductions in systolic blood pressure (23, 36, 40). Significant reductions in diastolic blood pressure were reported in fewer studies (23, 40). Meta-analysis showed a significant benefit with TCM interventions compared with controls (SMD −0.43, 95% CI −0.64 to −0.21; Z = 3.93, p < 0.0001). Moderate heterogeneity was identified (I² = 72%), which may be attributable to differences in intervention types, syndrome classifications, and blood pressure measurement methods.

**Figures 4b** and **5b** present funnel plots for the sleep and blood pressure outcomes, respectively. The sleep funnel plot showed an uneven distribution of studies around the pooled effect, whereas the blood pressure funnel plot showed a dispersion of smaller studies without a clear inverted-funnel structure. These findings indicate possible small-study effects. Publication bias for sleep outcomes was examined using Egger’s linear regression and Begg’s rank correlation tests (k = 7). Neither test reached statistical significance (Egger’s intercept = −9.21, 95% CI −23.20 to 4.79; t = −1.69, p = 0.152; Begg’s τ = −0.048, p = 1.000). Interpretation of these results is limited because statistical tests have low power when fewer than ten studies are available. Trim-and-fill analysis was not performed because only seven studies contributed to the sleep outcome pool. Funnel plot asymmetry, together with the limited number of studies, indicates that the pooled sleep estimate (SMD −2.21) should be interpreted with appropriate consideration of potential small-study effects.

**Figure 6:**
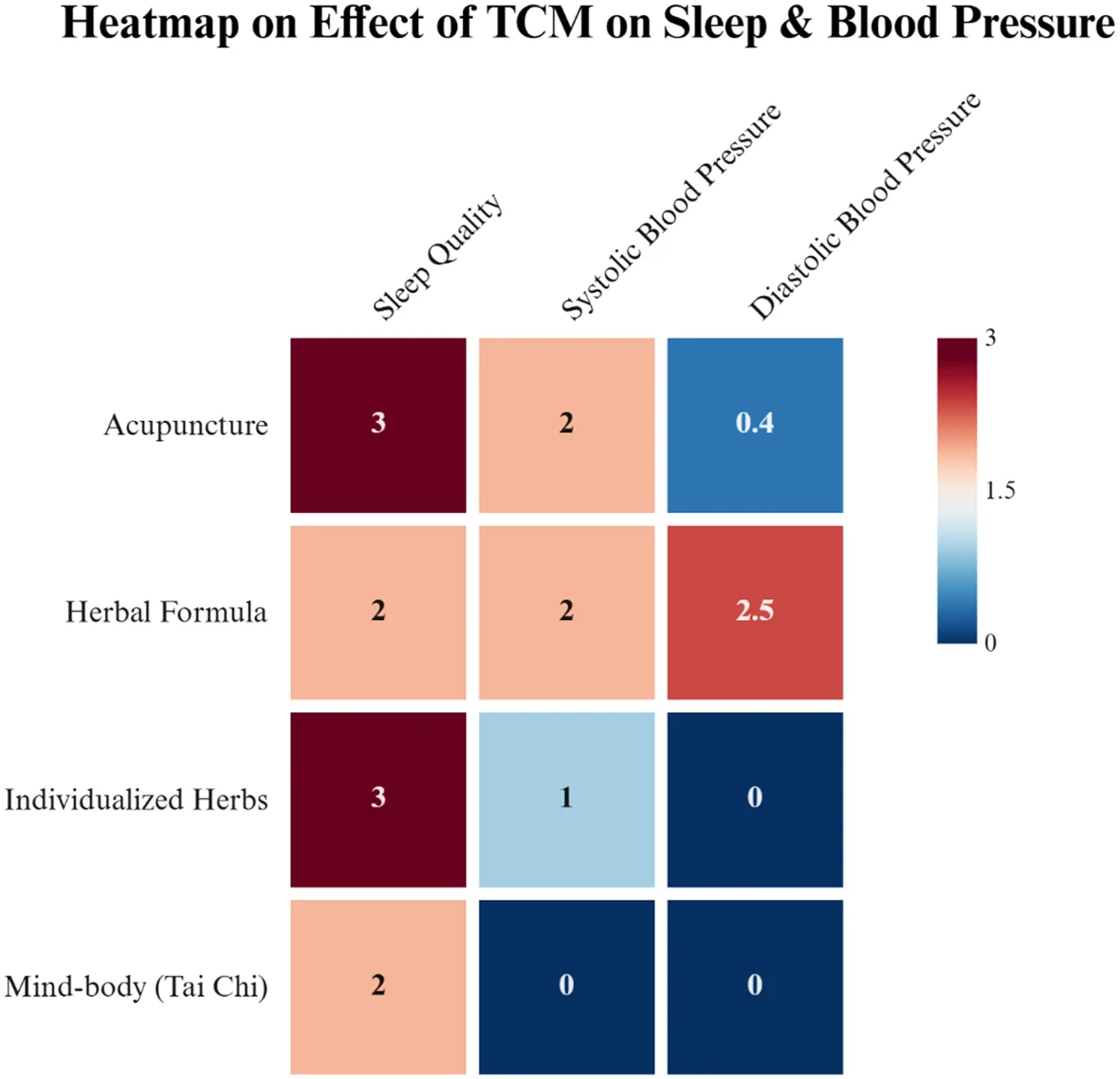
Heatmap of effect sizes for TCM interventions on sleep and blood pressure outcomes. Color intensity in the heatmap corresponds to the reported effect size magnitude for each outcome. The coding is as follows: Dark Red = 3 (Strongest Effect), Light Pink = 2 (Moderate Effect), Medium Blue = 2.5, Light Blue = 0.4 (Weak Effect), Light Green/White = 0 (No Apparent Effect). Numerical values within cells represent the specific effect size estimate for the respective TCM intervention and outcome measure.

**Figure 6** summarizes the relative effects of TCM interventions on sleep quality and blood pressure, with color intensity ranging from blue (0) to red (3). Acupuncture and individualized herbal therapy showed the greatest effects on sleep quality (3.0), followed by mind–body interventions (2.0). Herbal formulas demonstrated moderate effects on systolic blood pressure (2.0) and the largest effects on diastolic blood pressure (2.5). Individualized herbal therapy showed limited effects on systolic blood pressure (1.0) and no effect on diastolic blood pressure, while Tai Chi showed no measurable blood pressure effect. These findings are consistent with trial results reporting improved sleep outcomes with acupuncture and individualized herbal interventions, and greater blood pressure reduction with syndrome-specific herbal formulas. The figure highlights differences in treatment response between sleep and blood pressure outcomes and supports further evaluation of individualized treatment protocols.

### Subgroup analyses

Sleep outcomes showed a large pooled effect (SMD −2.21, 95% CI −3.26 to −1.16) with very high heterogeneity (I² = 97%). Subgroup analyses identified intervention modality, syndrome restriction status, and treatment duration as significant effect modifiers. Acupuncture produced a large effect with moderate heterogeneity (SMD −1.85, 95% CI −2.41 to −1.29; I² = 42%), whereas herbal medicine (SMD −2.67, 95% CI −4.89 to −0.45; I² = 98%) and mind-body interventions (SMD −2.90, 95% CI −3.60 to −2.20; I² = 0%) showed larger effect estimates. Studies requiring syndrome confirmation reported an SMD of −2.10 (95% CI −3.05 to −1.15; I² = 89%), compared with −2.45 (95% CI −4.20 to −0.70; I² = 99%) in studies without this requirement. Shorter interventions (<4 weeks) yielded greater benefits than longer treatments. High heterogeneity within most subgroups indicates that variability arose from a combination of clinical and methodological differences rather than a single identifiable factor (**Table 5**).

**Table 5:** Subgroup analysis of sleep quality outcomes (PSQI) according to intervention modality, syndrome restriction status, and treatment duration.

Subgroup variable	Category	No. of studies	Pooled SMD (95% CI)	I² (%)	Q-between	P-value
Intervention Modality					10.55	0.005
	Acupuncture	3	−1.85 (−2.41 to −1.29)	42		
	Herbal medicine	3	−2.67 (−4.89 to −0.45)	98		
	Mind-body intervention	1	−2.90 (−3.60 to −2.20)	—		
Syndrome Restriction Status					3.96	0.047
	Mandatory syndrome confirmation for enrolment	4	−2.10 (−3.05 to −1.15)	89		
	Optional/not reported	3	−2.45 (−4.20 to −0.70)	99		
Treatment Duration					4.26	0.039
	<4 weeks	3	−2.80 (−4.10 to −1.50)	95		
	4–8 weeks	3	−1.90 (−2.80 to −1.00)	78		
	>8 weeks	1	−1.64 (−2.81 to −0.47)	—		

## Discussion

The meta-analysis reported pooled improvement in sleep quality (SMD −2.21, 95% CI −3.26 to −1.16) and blood pressure control (SMD −0.43, 95% CI −0.64 to −0.21) with syndrome-informed TCM interventions in adults with comorbid hypertension and insomnia (1). Interpretation of these estimates requires caution because none of the included trials applied individualized syndrome differentiation as used in routine TCM practice (17, 37). The included studies used fixed, syndrome-labeled protocols or stratified allocation without individual-level treatment modification, which limits inference about pattern-based care (20, 37). The pooled findings reflect fixed treatment protocols for predefined TCM syndromes and may not be fully generalizable to routine clinical TCM practice, where herbal prescriptions and acupuncture treatments are individualized and modified in response to changes in the patient’s syndrome over the course of treatment.

Pattern-restricted signals were reported in selected analyses, with Gastrodia–Uncaria granules reducing systolic blood pressure only among participants classified with liver-yang hyperactivity (37). Population studies have reported higher odds of insomnia among specific TCM constitutions, including qi-depression and phlegm-dampness (2, 30). These observations indicate that syndrome classification facilitates outcome stratification. Current trials evaluate predefined syndrome-based protocols rather than patient-specific treatment selection. The review included comorbid hypertension-insomnia and single-condition populations receiving syndrome-differentiated TCM interventions. Clinical heterogeneity was examined using pre-specified subgroup analyses for comorbid and single-condition cohorts.

This systematic review and meta-analysis showed improved sleep quality and better blood pressure control with syndrome-informed TCM interventions in adults with comorbid hypertension and insomnia (1). Syndrome differentiation in the included trials was applied through syndrome-specific protocols or restriction to particular syndromes rather than individualized treatment selection used in routine practice (17). Twelve studies provided fixed interventions to all participants, whereas four studies enrolled a single syndrome group and administered the same treatment throughout, such as Jiaotaiwan granules for heart–kidney disharmony (20, 37). Therefore, the pooled findings represent outcomes of predefined syndrome-based treatment protocols and cannot determine the effect of individualized syndrome-guided treatment.

The Gastrodia–Uncaria granule trial reported a reduction in 24-hour ambulatory systolic blood pressure only among participants classified with liver-yang hyperactivity syndrome with no effect in non-liver-yang subgroups (37). Xia et al. (20) reported that Jiaotaiwan granules reduced PSQI scores in patients with heart–kidney disharmony (mean reduction 1.64 points, 95% CI −2.81 to −0.47; P = 0.006) with an increase in serum melatonin (P = 0.004), whereas polysomnographic indices did not change, thus subjective sleep improvement occurred without parallel objective sleep architecture changes. Choi et al. (47) reported that Huanglian-Jie-Du granules reduced ISI scores in patients classified with heat or fire patterns, with greater improvement in pattern-related symptoms than in general somatic complaints.

Han et al. (30) reported higher odds of insomnia among individuals classified with qi-depression (OR 2.12, 95% CI 1.45–3.12) and phlegm-dampness constitutions (OR 1.97, 95% CI 1.37–2.85). Using data from the Tianning Cohort, Chen et al. (2) found that poor sleep quality (PSQI ≥5) increased the prevalence of hypertension by 17% after adjustment for demographic, lifestyle, metabolic, and anthropometric variables (OR 1.17, 95% CI 1.01–1.36; P = 0.042). Subjective sleep quality, sleep latency, and sleep disturbances were associated with hypertension, whereas sleep duration was not (2, 3). The present meta-analysis evaluated whether syndrome-specific TCM interventions improved both sleep and blood pressure. The pooled sleep effect exceeded the minimal important difference of 2.8 PSQI points (48), whereas the pooled systolic blood pressure effect remained below the 5-mmHg threshold associated with cardiovascular risk reduction (3).

Most participants presented with mixed constitutions, commonly qi-deficiency with blood-stasis or phlegm-dampness with damp-heat, highlighting the complexity of clinical populations (19, 30). Pan et al. (9) reported increased Lactobacillus abundance and reduced IL-6 following TCM intervention, accompanied by improvements in sleep architecture and inflammatory status. Jin et al. (27) showed that Gastrodia–Uncaria reduced oxidative stress through Nrf2/HO-1 activation, a mechanism relevant to sleep maintenance and nocturnal blood pressure regulation. Related findings on gut microbial GABA production (49), circadian regulation of Nrf2 through BMAL1/E-box interactions (50, 51), enhancement of GABAergic signaling by TCM interventions (52), provide biological plausibility for concurrent improvements in sleep and blood pressure. Subgroup analyses identified intervention modality, syndrome restriction status, and treatment duration as sources of variation. Heterogeneity persisted within subgroups due to differences in syndrome presentation, comparator selection, and baseline disease severity (17, 22, 53). Component analyses identified subjective sleep quality, sleep latency, and sleep disturbances as contributors, whereas sleep duration showed no association, thus implicating sleep fragmentation and hyperarousal pathways, including sympathetic activation, hypothalamic–pituitary–adrenal axis dysregulation, and endothelial dysfunction (2, 3). This explains why TCM interventions directed at sleep-related neurophysiological regulation yield concurrent effects on sleep and blood pressure.

Experimental and translational studies have described biological mechanisms relevant to sleep regulation and blood pressure control. Pan et al. (9) reported that herbal formulas and acupuncture modified gut microbiota composition, increasing Lactobacillus and Bifidobacterium abundance while reducing circulating IL-6 and TNF-α. These changes are associated with sleep quality and vascular function. Jin et al. (27) demonstrated that Gastrodia–Uncaria reduced oxidative stress through activation of the Nrf2/HO-1 pathway and suppression of NADPH oxidase, mechanisms involved in nocturnal blood pressure regulation and sleep maintenance. TCM interventions may affect the autonomic activity, cortisol rhythms, and neurotransmitter systems involved in sleep regulation, unlike antihypertensive drugs that primarily target vascular resistance (3, 9, 27). Clinical findings also indicate that better sleep is accompanied by lower blood pressure. Li et al. (3) reported a 4.2-mmHg greater reduction in systolic blood pressure among patients whose insomnia improved compared with non-responders (P < 0.001). Wang F et al. (43) found that Yangxue Qingnao Pills combined with amlodipine reduced blood pressure variability and improved insomnia response rates compared with amlodipine alone in patients with blood deficiency and liver-yang hyperactivity (P < 0.05).

TCM and acupuncture improved sleep quality, blood pressure regulation, and health-related quality of life in patients with hypertension and insomnia (25). Herbal, acupuncture, and complementary therapies reduced insomnia symptoms with accompanying improvements in hemodynamic parameters (28, 29, 54, 55). Prescriptions developed for heart–kidney disharmony and liver-yang hyperactivity improved both sleep and blood pressure outcomes, indicating variation in response according to syndrome classification (46, 47, 56, 57). Adverse event rates were comparable between intervention and comparator groups (26, 58, 59). Jiao-tai-wan and Shumian capsule were well tolerated in hypertensive adults with insomnia and may serve as treatment options when the use of sedative medications requires caution (2, 24, 57).

The pooled results showed clinically meaningful improvement in sleep quality, while blood pressure reduction remained below the threshold associated with cardiovascular risk reduction. Direct comparison of fixed and individualized treatment protocols requires further investigation (3, 17, 22, 53, 60). TCM interventions were generally well tolerated, with adverse event rates similar to those of control groups (RR 1.08, 95% CI 0.82–1.42) (1, 28). Benzodiazepine use has been associated with dependence, cognitive impairment, and falls in older adults with hypertension (35). The frequent coexistence of insomnia and hypertension supports evaluation of treatment strategies addressing both sleep quality and blood pressure. TCM may serve as an adjunctive therapeutic option.

The included studies used fixed treatment protocols within predefined syndrome categories rather than individualized syndrome differentiation used in routine TCM practice (37, 39). No included study directly compared individualized treatment with fixed protocols in patients diagnosed with both hypertension and insomnia. Subgroup-specific effects were observed, with Gastrodia–Uncaria granules reducing systolic blood pressure in participants with liver-yang hyperactivity (37), and Jiaotaiwan granules reducing PSQI scores in heart–kidney disharmony (20). These findings suggest clinical benefits within defined syndrome categories but do not establish whether individualized treatment provides superior outcomes. Most included trials evaluated treatment for only 4–8 weeks. Future studies with longer treatment and follow-up periods are needed to determine whether improvements in sleep quality and blood pressure are maintained and to evaluate the long-term safety of TCM interventions. Future studies should also compare individualized and fixed TCM protocols in comorbid hypertension and insomnia using validated syndrome classification, ambulatory blood pressure monitoring, and actigraphy (17, 22, 53). The findings indicate that syndrome-informed TCM interventions improve sleep quality and blood pressure and could serve as a useful adjunctive treatment approach.

### Strengths and limitations

This meta-analysis followed a predefined protocol and included only RCTs. Quantitative synthesis used standardized effect estimates with formal heterogeneity assessment. Methodological quality was evaluated using the ROB 2 tool and the JADAD Scale. Syndrome-specific analyses helped the evaluation according to defined TCM classifications. Individualized syndrome differentiation was not consistently applied in clinically heterogeneous populations. Most studies depended on subjective sleep assessments and did not report long-term cardiovascular outcomes. Publication bias cannot be definitively ruled out because fewer than 10 studies were available for several pooled outcomes, which limited the statistical assessment of publication bias. Generalizability is reduced due to restricting the search to English-language databases, as major Chinese databases were not searched.

## Conclusion

This systematic review and meta-analysis show that syndrome-specific TCM interventions improve sleep quality and reduce blood pressure in adults with comorbid hypertension and insomnia. The results show concurrent changes in sleep disturbance and hemodynamic measures, which commonly coexist in this population. The use of TCM as an adjunct to antihypertensive therapy could be considered in patients with persistent insomnia. In clinical practice, patients with hypertension should be screened for insomnia using validated tools, followed by identification of TCM syndromes using standard diagnostic criteria. The treatment procedures can then be selected according to the syndrome, for example Jiaotaiwan for heart–kidney disharmony or Gastrodia–Uncaria for liver–yang hyperactivity, along with antihypertensive drugs. It is essential to monitor sleep scores and blood pressure at regular intervals at home, as this can guide treatment decisions. However, studies that compare individualized TCM with standard protocols in patients with both conditions are required to define its clinical role.

## Data Availability

The original contributions presented in the study are included in the article/supplementary material. Further inquiries can be directed to the corresponding authors.
